# Characterisation of a *puc*BA deletion mutant from *Rhodopseudomonas palustris* lacking all but the *puc*BA_d_ genes

**DOI:** 10.1007/s11120-017-0386-7

**Published:** 2017-05-31

**Authors:** June Southall, Sarah L. Henry, Alastair T. Gardiner, Aleksander W. Roszak, William Mullen, Anne-Marie Carey, Sharon M. Kelly, Claire Ortmann de Percin Northumberland, Richard J. Cogdell

**Affiliations:** 1 Institute of Molecular Cell and Systems Biology, University of Glasgow, 126 University Place, Glasgow, G12 8TA Scotland, UK; 2 Biomedical Engineering, School of Engineering, University of Glasgow, 126 University Place, Glasgow, G12 8TA Scotland, UK; 3 Institute of Cardiovascular and Medical Sciences, University of Glasgow, 126 University Place, Glasgow, G12 8TA Scotland, UK; 4 Center for Innovations in Medicine, The Biodesign Institute, Arizona State University, 1001 S. McAllister Ave., Tempe, AZ 85287-5001 USA

**Keywords:** Photosynthesis, Purple bacteria, *Puc* genes, Light-harvesting, LH2

## Abstract

*Rhodopseudomonas palustris* is a species of purple photosynthetic bacteria that has a multigene family of *puc* genes that encode the alpha and beta apoproteins, which form the LH2 complexes. A genetic dissection strategy has been adopted in order to try and understand which spectroscopic form of LH2 these different genes produce. This paper presents a characterisation of one of the deletion mutants generated in this program, the *puc*BA_d_ only mutant. This mutant produces an unusual spectroscopic form of LH2 that only has a single large NIR absorption band at 800 nm. Spectroscopic and pigment analyses on this complex suggest that it has basically a similar overall structure as that of the wild-type HL LH2 complex. The mutant has the unique phenotype where the mutant LH2 complex is only produced when cells are grown at LL. At HL the mutant only produces the LH1-RC core complex.

**Electronic supplementary material:**

The online version of this article (doi:10.1007/s11120-017-0386-7) contains supplementary material, which is available to authorised users.

## Introduction

Light is never constant in the natural environment. Purple photosynthetic bacteria have evolved a number of different phenotypic responses that enable them to adapt to changes in light intensity. Purple bacteria are able to regulate the amount of photosynthetic membranes, called intracytoplasmic membranes (ICM), present within their cells to allow them to absorb a sufficient number of photons to grow photosynthetically. The ICM within the wild-type purple bacterial cells are present in different morphologies, such as vesicular, tubular or lamellar membranes (Remsen 1978). In high-light (HL) conditions the amount of ICM is small and under low-light (LL) conditions the amount is much greater (Varga and Staehelin 1983). Most purple photosynthetic bacteria have ICM in which photosynthetic units (PSU) contain light-harvesting 1-reaction centre ‘core’ complexes (LH1-RC) and light-harvesting 2 (LH2) complexes (Robert et al. 2003; Cogdell et al. 2004, 2006; Law et al. 2004). The number of PSUs per cell and the ratio of LH2 to ‘core’ complexes vary depending on the light intensity at which the cells are grown (Aagaard and Sistrom 1972). Recent AFM studies, especially by the groups of Hunter and Scheuring, have shown that the ICM are always rather ‘full’ (Bahatyrova et al. 2004; Scheuring and Sturgis 2005; Sturgis et al. 2009) and the ratio of LH2 to ‘core’ complexes increases as the light intensity decreases. However, in some species there is a further, more subtle change that takes place within the ICM in response to growth at different light intensities. Some species of purple photosynthetic bacteria are also able to alter the spectroscopic form of LH2 present depending on the incident light intensity (Hayashi et al. 1982a, b; Evans et al. 1990; Gardiner et al. 1993; Carey et al. 2014).

The α- and β-apoproteins that oligomerise to form LH2 complexes are encoded by the *puc*BA genes (Youvan and Ismail 1985; Kiley and Kaplan 1987; LeBlanc and Beatty 1993). The different spectroscopic forms of LH2 result from the expression of different *puc* genes and the incorporation of their respective polypeptides into new LH2 complexes with altered near infra-red (NIR) absorption bands (Brunisholz and Zuber 1988). *Rhodopseudomonas* (*Rps*.) *palustris*, for example, has a multigene family of five *puc*BA gene pairs encoding the LH2 apoproteins (Tadros and waterkamp 1989). The five putative *puc*BA gene pairs in the *Rps. palustris* genome are called *puc*BA_a_ to *puc*BA_e_ respectively, with *puc*BA_c_ presumed to be a pseudogene that is unable to form a functional LH2. Interestingly as more genome sequences from purple bacterial species become available, the presence of multiple *puc*BA genes appears to be the rule rather than the exception. These multiple *puc*BA gene pairs can be widely separated within the genome and can also be transcribed from different DNA strands. The only species definitively known to have a single *puc*BA gene pair is *Rubrivivax gelatinosus* (Steunou et al. 2004).

The presence of a multigene family for LH2 can lead to an inherent added structural complication for this complex. Unlike the LH2 complex from *Rhodoblastus (Rbl.) acidophilus* (previously *Rps acidophila*) strain 10050 where the apoprotein composition of the LH2 rings is homogeneous (McDermott et al. 1995), LH2 rings in *Rps. palustris* are heterogeneous with individual rings having α- and β-apoproteins present that are encoded by different *puc*BA genes (Tadros et al. 1993; Larimer et al. 2004). The spectroscopic form of LH2 in these heterogeneous rings depends upon the exact disposition of the different types of apoprotein around the ring. The current working hypothesis, derived from mass spectrometry analyses, is that the HL LH2 from *Rps. palustris* contains mainly the polypeptides from *puc*BA_a_ and *puc*BA_b_ with none from *puc*BA_d_ and that the LL LH2 contains mostly polypeptides from *puc*BA_d_ with minor amounts from *puc*BA_a_ and *puc*BA_b_ (Brotosudarmo et al. 2009). The complication induced by the presence of multiple types of α- and β-polypeptides within a single LH2 complex ring makes it difficult, in the absence of high-resolution structures, to understand the exact contribution each *puc*BA gene pair product makes to the overall spectroscopic form of LH2. This depends not only on the amino acid compositions of the apoproteins present (Fowler et al. 1992) but also on how they are arranged relative to each other in the individual rings (Brotosudarmo et al. 2011).

In order to try and explicitly resolve this fundamental question we have adopted a genetic dissection strategy and are in the process of creating *puc*BA deletion mutants in *Rps. palustris* for all the possible combinations. Each of these mutants are being investigated in turn, under HL and LL conditions, to try and understand how the deletions affect the regulation of LH2 expression, the apoprotein composition of the complexes and the spectroscopic phenotype.

The quadruple mutant of *Rps. palustris* characterised in this paper has had the *puc*BA_a_, *puc*BA_b_, *puc*BA_c_ and *puc*BA_e_ gene pairs deleted (Δ*puc*BA_abce_) so that only the *puc*BA_d_ gene pair remains in the genome. The resulting LH2 complex produced by this mutant has some totally unprecedented phenotypic and spectroscopic properties and these are described below.

## Materials and methods

### Bacterial culture conditions

Cells of *Rps. palustris* were cultured in C-succinate media in flat, glass bottles at 30 °C under anaerobic conditions with illumination provided by incandescent bulbs (Gest and Bose 1963). HL conditions were 50 µmol photons/s/m^2^ and LL conditions were 3 µmol photons/s/m^2^. All initial cultures were started from a single colony previously confirmed by PCR to ensure no cross contamination between the wild-type and the deletion strains. Once the cells had grown, and were fully adapted to the light intensity, they were harvested by centrifugation, washed in 20 mM MES, 100 mM KCl, pH 6.8 and then either used immediately or stored at −20 °C until required (Evans et al. 1990).

### Construction of the deletion strain

All cloning methods, unless otherwise stated, were carried out as performed in Henry et al. (2016). The genomic DNA sequence for *Rps. palustris* was obtained from the NCBI database *Rps. palustris* CGA009 and all Genbank numbers for *puc*BA genes are listed in Supplementary Table 1. To generate the deletion Δ*puc*BA_abce_ strain the *puc*BA genes were amplified from genomic DNA extracted from cultured cells. Deletions for *puc*BA_a_ and *puc*BA_c_ were generated by the splice overlap extension PCR (Henry et al. 2016). In short, the central part of the *puc*BA gene was removed by amplifying DNA upstream and downstream of the *puc*BA gene and then annealed, leaving only the first and last two codons of the *puc*BA wild-type gene. Sequences for all oligonucleotides are listed in Supplementary Table 2. Deletions in *puc*BA_b_ and *puc*BA_e_ genes were generated with the addition of streptomycin and chloramphenicol resistance genes, respectively. These genes were amplified from vectors pSW25 and pSW23, inserted into the cloned *puc*BA_b_ and *puc*BA_e_ genes using standard molecular cloning techniques (Sambrook and Russell 2001). All deleted *puc*BA constructs were ligated into the suicide vector pK18mobsacB (supplied as a gift from Judy Armitage, Oxford) (Schafer et al. 1994). Each *puc*BA gene was deleted individually to produce a Δ*puc*BA_abce_ quadruple strain carrying only the *puc*BA_d_ gene.

The pK18mobsacB plasmids containing disrupted *puc*BA genes were transferred into *Rps. palustris* cells by conjugation from *E. coli* S17λ pir. Single colonies with kanamycin resistance were grown and serial dilutions plated on C-succinate agar minus casamino acids were supplemented with 10% (w/v) sucrose and incubated for 3–5 days at 30 °C. Single colonies were transferred onto duplicate grid screens: one plate containing only succinate and the other plate containing succinate and kanamycin. Colonies growing on succinate but not on succinate–kanamycin were analysed by PCR to detect correct recombination of *puc*BA gene into the *Rps. palustris* genome and confirmed by sequencing.

### Protein purification

*Rps. palustris* cells were re-suspended in 20 mM Tris–Cl pH 8.0, homogenised with the addition of ~100 µg of DNase and a few mgs of MgCl_2_ and disrupted by two passages through a French Pressure cell (~15,000 psi). The photosynthetic membranes were immediately pelleted by ultracentrifugation (180,000 g, 90 min, 4 °C). The supernatant was discarded and the membranes were gently re-suspended with 20 mM Tris–Cl pH 8.0 and adjusted to an optical density (OD) at 850 nm of 50 cm^−1^. The re-suspended membranes were solubilised at room temperature for 120 min with 2% *N,N*-dimethyldodecylamine *N*-oxide (LDAO) and then centrifuged to remove any un-solubilised material. The solubilised membrane fraction was fractionated using stepwise sucrose density centrifugation (150,000 g, 4 °C, 16 h). The LH2 complex band was collected from the gradient and loaded on to a Q-Sepharose (GE Healthcare) anion exchange column. The LH2 was washed on the column with detergent—Tris buffer solution and eluted with increasing concentrations of NaCl. However, the particular detergent was dependent upon the experiment. The LH2 sample was either retained in 0.1% LDAO or exchanged into 0.02% *n*-dodecyl-β-d-maltoside (DDM) or 0.15% *n*-decyl-β-d-maltoside (DM). The LH2 was then further purified by gel filtration using a Sephadex-200 column. The resulting fractions were collected, assayed spectrophotometrically (using the ratio of A_800_/A_260_) and the best pooled and concentrated to the desired absorbance. The final pool was checked using SDS-PAGE to ensure that protein was pure.

### SDS-PAGE gel electrophoresis

LH2 samples for SDS-PAGE were run using the Invitrogen NuPAGE electrophoreses system. Samples were reduced and heated at 70 °C for 10 min before loading onto a 4–12% MES Tris-Glycine gel and run at 200 V for approx. 35 min. The gel was stained with SimplyBlue (Invitrogen) or silver ions as appropriate.

### Electron microscopy of whole cells

Bacterial cell suspensions were washed in phosphate buffered saline before being initially fixed with 2.5% glutaraldehyde, 2% formaldehyde, 0.1 M sodium cacodylate (1 h).

Suspensions were rinsed with 0.1 M sodium cacodylate buffer and then post-fixed in 1% osmium tetroxide buffer (1 h) followed by washing with distilled water. The bacterial suspensions were spun down in agarose, diced into smaller 1–2 mm pieces and stained with 0.5% uranyl acetate.

The samples were briefly washed in distilled water to remove any excess stain and dehydrated through a series of ethanol washes, dried and treated with propylene oxide before being left overnight in a 1:1 mixture of propylene oxide and 502/812 EPON resin. During the course of the following day several changes of pure EPON resin were performed and the samples were embedded in fresh resin and polymerised at 60 °C for 24–48 h. Ultrathin section (50 nm thickness) were cut using a Leica Ultracut UTC and a Drukker diamond knife. The bacterial resin sections were collected onto 300 mesh Formvar-coated copper support grids and contrast stained with 2% methanolic uranyl acetate and Reynolds lead citrate. All sample processing was performed at room temperature. The bacterial cell sections were viewed on a FEI Tecnai T20 TEM running at 200 kV. The images were captured using a GATAN Multiscan camera 794 and processed using GATAN digital imaging software.

### Peptide analysis by mass spectrometry

The α- and β polypeptides were extracted from the purified LH2 at A_850_ = 100 cm^−1^ complexes and prepared for mass spectrometry as previously described (Parkes-Loach et al. 1988). Samples were analysed on a Dionex Ultimate 3000 RSLS nano flow system (Dionex, Camberly UK). After loading onto a Dionex 0.1 × 20 mm 5 µm C18 nano trap column at a flowrate of 5 µl/min in 98% 0.1% formic acid and 2% acetonitrile, sample was eluted onto an Acclaim PepMap C18 nano column 75 µm × 15 cm, 2 µm 100 Å at a flow rate of 0.3 µl/min. The trap and nano flow column were maintained at 35 °C. The samples were eluted with a gradient of solvent A: 98% 0.1% formic acid, 2% acetonitrile verses solvent B: 80% acetonitrile, 20% 0.1% formic acid starting at 35% B for 5 min rising to 65% B after 60 min. The column was then washed and re-equilibrated prior to the next injection.

The eluant was ionised using a Proxeon nano spray ESI source operating in positive ion mode into an Orbitrap Velos FTMS (Thermo Finnigan, Bremen, Germany). Ionisation voltage was 2.6 kV and the capillary temperature was 250 °C. The mass spectrometer was operated in MS/MS mode scanning from 700 to 1800 amu. The top five multiply charged ions were selected from each scan for MS/MS analysis using HCD at 50% collision energy. The resolution of ions in MS1 was 60,000 and 7,500 for MS2.

MS and MS/MS data files were searched, in this case, against the Uniprot *Rps. palustris* database using SEQUEST (using Thermo Proteome Discoverer™), with no enzyme specified. No static modification was set and the oxidation of methionine and proline (M, P) were set as variable modifications. The peptide mass tolerance was set at ±10 ppm, the fragment mass tolerance set at ±0.05 Da and a maximum of one missed cleavage was allowed for. Peptide data were extracted using high peptide confidence, mass deviation of ±5 ppm and top one peptide rank filters.

### Circular dichroism measurements

All circular dichroism (CD) spectra were measured at room temperature using a Jasco J-810 spectropolarimeter and a 0.2 cm path length quartz cuvette with LH2 complex at* A*_800_ = 2.0. The NIR photomultiplier parameters were set as follows; data pitch 0.2 nm, slit width 60 µm, response 1 s. Two scans for each sample were collected at a speed of 20 nm/min.

### Determination of the LH2 bacteriochlorophyll:carotenoid ratio

The LH2 bacteriochlorophyll (Bchl) *a*:carotenoid ratio was measured by extracting the pigments using acetone and methanol (7:2 v/v) in the dark. Three to four separate preparations of each type of LH2 complex were extracted with two to four replicates. Samples were centrifuged at 3000 g for 3 min in Pyrex glass tubes and the absorption spectrum of the extract was recorded with a UV-1700 UV–VIS Shimadzu spectrophotometer in a 1 ml cell. The molar ratio of Bchl to carotenoid in the complexes can be calculated assuming a* ɛ* = 76/mM/cm for Bchl (Clayton 1966) and 160/mM/cm for the carotenoids (Britton 1985) present. The Bchl:carotenoid ratio was determined from the ratio of the absorption maximum of the carotenoids and the peak of the Bchl Q_y_ band at 772 nm.

### Crystallisation

The LH2 complex isolated from the Δ*puc*BA_abce_ mutant in 20 mM Tris–Cl pH 8.0, 0.02% DDM was concentrated to A_800_ = 100 and the amphiphile molecules benzamidine, spermidine or 1,2,3-heptanetriol (HPT) were dissolved gently in the LH2 solution to give a final concentration of 1.0% w/v. The LH2 samples were placed into initial crystallisation trials using a Cartesian Honeybee 8+1 crystallisation robot together with the HT Sparse-matrix screens MemGold, MemGold 2 and MemStart/Sys purchased from Molecular Dimensions.

For crystallisation trials in 24-well plates (Cryschem, NBiS Biologicals) the following crystallisation protocol was used. The mutant LH2 in 20 mM Tris–Cl pH 8.0, 0.15% DM was adjusted to A_800_ = 100 and the amphiphile HPT added and dissolved gently to give a final concentration of 3.0% w/v. This solution was then mixed gently in a 1:1 ratio with a reservoir solution of 21–19% Polyethylene glycol (PEG) 600, 50 mM CaCl_2_, 20 mM Tris–Cl pH 8.0, microfuged and the supernatant pipetted (20 µl) into the wells of the plate. Plates were incubated at 16 °C and crystals grew by vapour diffusion in 6 weeks. Crystals were tested on our home-source X-ray generator and the best crystals were sent to the Diamond Light Source (DLS) near Oxford for data collection.

## Results

**Fig. 1:**
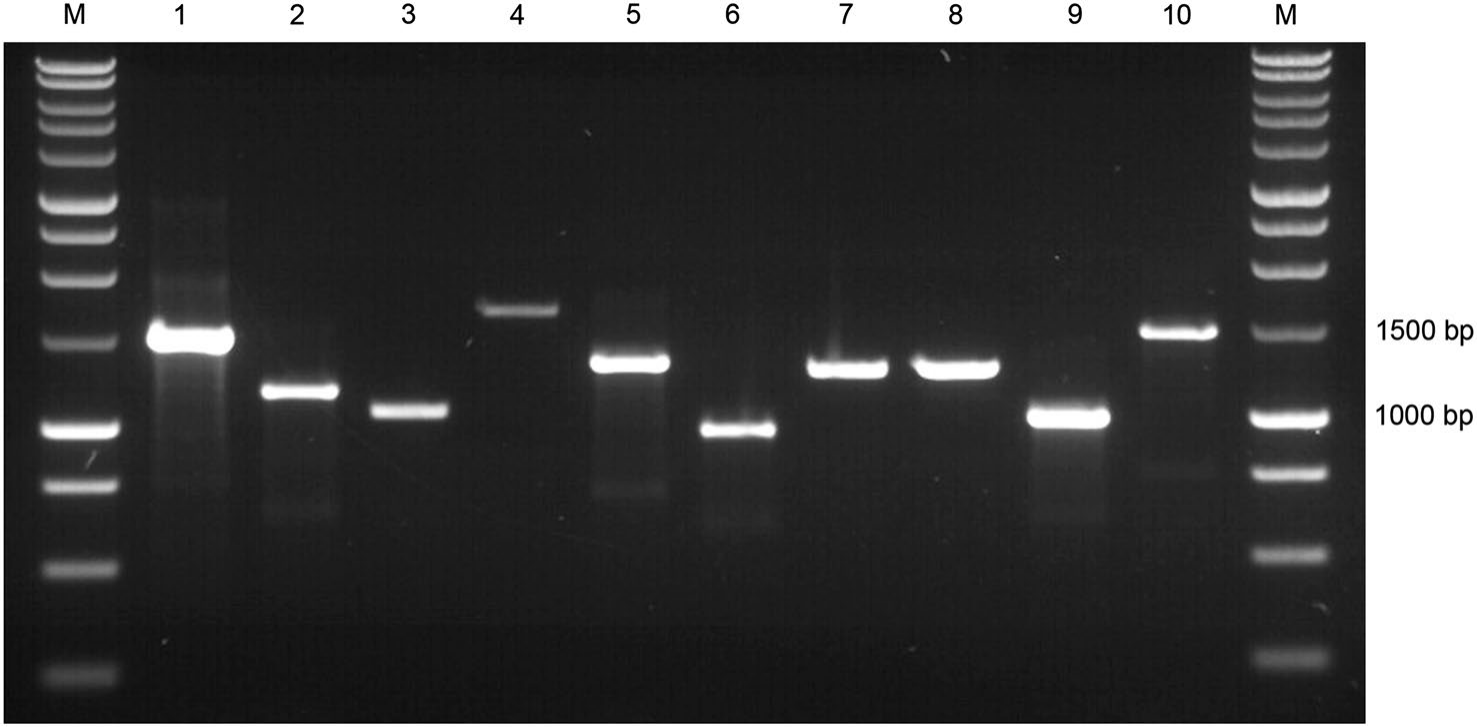
PCR analysis of the five *pucBA* gene pairs from the *Rps. palustris* Δ*puc*BA_abce_ quadruple mutant and the wild-type strain. The two outside lanes are M-Promega 1 kb ladder marker and lanes 1–10 contain PCR fragment sizes a follows from (*1*) wild-type *puc*BA_a_ 1498 bp, (*2*) Δ*puc*BA_a_ 1168 bp, (*3*) wild-type *puc*BA_b_ 1047 bp, (*4*) Δ*puc*BA_b_ 1661 bp, (*5*) wild-type *puc*BA_c_ 1280 bp, (*6*) Δ*puc*BA_c_ 930 bp, (*7*) wild-type *puc*BA_d_ 1246 bp, (*8*) Δ*puc*BA_d_ 1246, (*9*) wild-type *puc*BA_e_ 1014 bp and (*10*) Δ*puc*BA_e_ 1488. The different sizes of the respective wild-type and deleted *puc*BA PCR bands from all gene pairs except *puc*BA_d_, indicate that only this gene pair is present in genomic DNA. The primer sequences are listed in Supplementary Table 3. All bands were excised and confirmed by sequencing

In order to confirm that the Δ*puc*BA_abce_ mutant contained only the *puc*BiA_d_ gene pair, PCR analysis of the five *puc*BA genes was performed at the start and the end of each growth experiment on extracted genomic DNA. Primers for each *puc*BA gene pair were designed outside the start and stop codons for both the wild-type and recombinant strains (see Supplementary Table 3). The ∆*puc*BA_a_ and ∆*puc*BA_c_ mutants were generated by deleting the gene through splice overlay extension PCR and, therefore, had 320 and 350 bp respectively deleted from the middle of the gene pair. As a result, the PCR band for these mutated, non-functional gene pairs is smaller than the corresponding wild-type gene pair, shown in Fig. 1, lanes 1, 2 and 5, 6 respectively. The *puc*BA_b_ and *puc*BA_e_ genes have been disrupted by the insertion of streptomycin and chloramphenicol genes, respectively, thereby, generating larger constructs than the corresponding wild-type *puc*BA gene shown in Fig. 1, lanes 3, 4 and 9, 10 respectively. Only the *puc*BA_d_ gene pair remains intact in the quadruple deletion mutant, Fig. 1, lanes 7, 8.

**Fig. 2:**
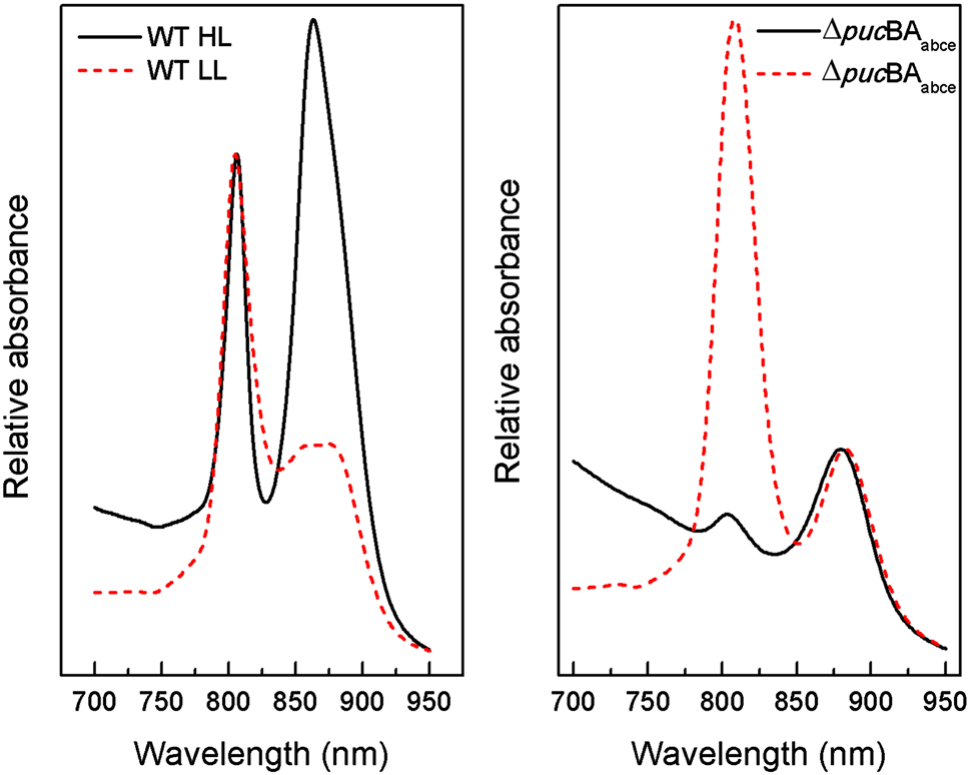
Absorption spectra of *Rps. palustris* membranes from HL (*black*) and LL (*dash*) grown cells from wild-type (*left*) and the ∆*puc*BA_abce_ mutant (*right*). In each case the membranes were re-suspended in 20 mM Tris–Cl pH 8.0. The spectra of the wild-type membranes were normalised at 800 nm and those of the ∆*puc*BA_abce_ mutant were normalised at 875 nm

*Rps. palustris* wild-type and the quadruple Δ*puc*BA_abce_ deletion mutants were grown at HL and LL and the NIR absorption spectra of membranes are shown in Fig. 2. The absorption spectra of wild-type membranes show the well described changes in the spectroscopic form of LH2, going from the standard LH2 under HL growth conditions (the high B850 form) to the low B850 form of LH2 under LL growth conditions (Brotosudarmo et al. 2011). In both conditions the LH1 absorption band is also present as a shoulder at about 875 nm. The absorption spectra of membranes prepared from the quadruple deletion mutant show dramatic differences compared to the wild type. The deletion mutant grown under HL conditions contains no LH2 complexes as the absorption spectrum only shows the presence of LH1-RC ‘core’ complexes. In this case the small peak at 803 nm originates from the RC. The absorption spectrum for membranes of Δ*puc*BA_abce_ deletion cells grown at LL show that an LH2 complex is now made but one that is very unusual. In this case the LH2 complex only features a single large absorption band in the NIR at 808 nm.

**Fig. 3:**
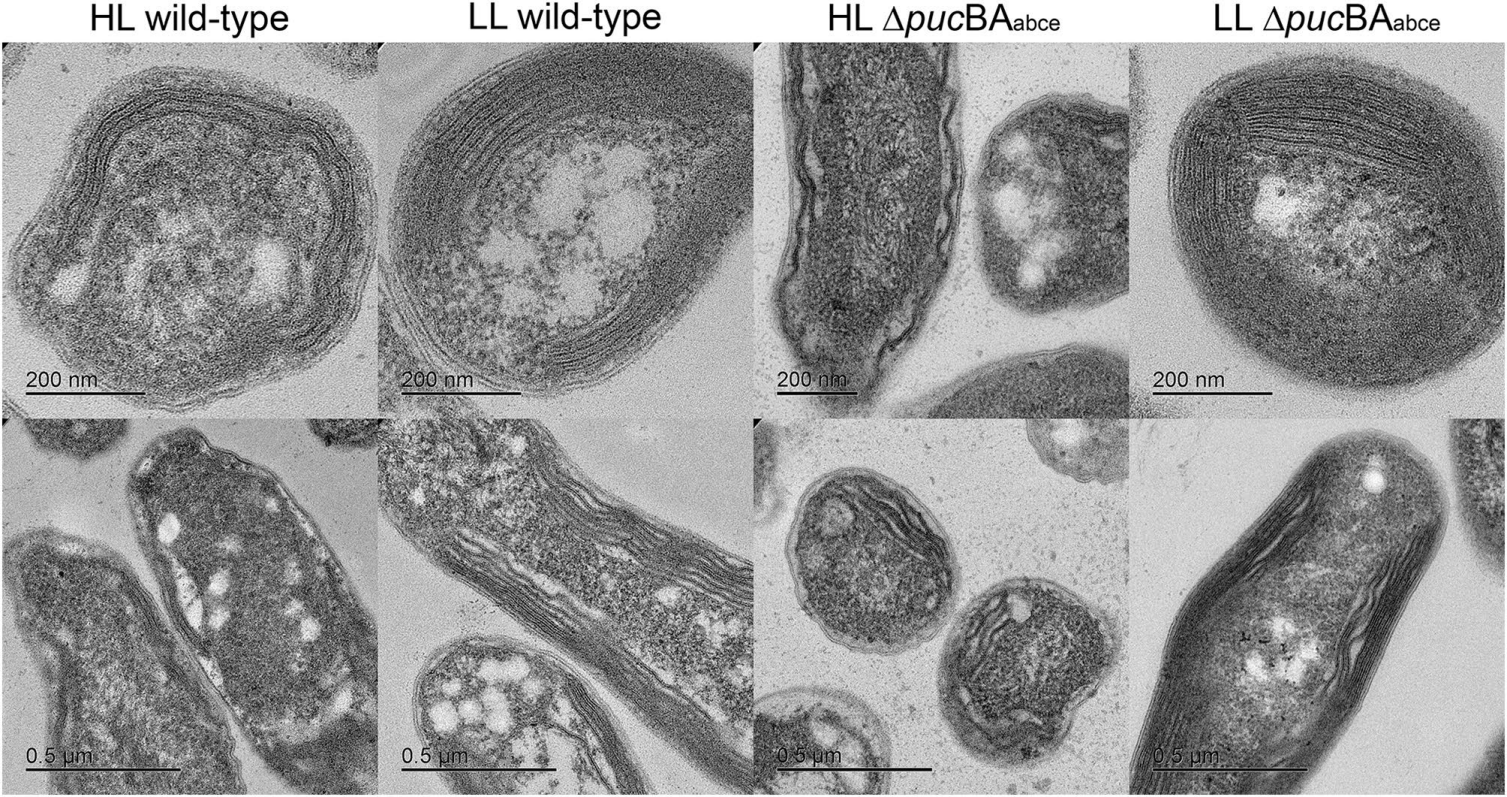
EM thin sections of a representative sample of cells grown at HL and LL of both the wild-type and the quadruple mutant. In each case the thin sections are shown at two different magnifications. The large white intracellular inclusions are stores of polyhydroxybutyrate

In some species of purple photosynthetic bacteria, such as *Rhodobacter (Rba.) sphaeroides*, the shape of their ICM changes in mutants that no longer synthesise LH2 complexes (Kiley et al. 1988; Qian et al. 2008). The wild-type *Rba. sphaeroides* cells have vesicular ICM whereas the mutants that only have LH1-RC ‘core’ complexes also have large tubular ICM. To investigate whether the shape of the ICM from *Rps. palustris* changes when only LH1-RC complexes are present in the thin sections of wild-type and Δ*puc*BA_abce_ cells grown at both HL and LL have been examined using electron microscopy. A selection of typical images for each cell type is presented in Fig. 3. In each case the micrographs of the thin sections have been recorded at two different magnifications. The wild-type cells show the typical result that the ICM are located around the periphery of the cells and the extent of the membranes depends on the light intensity at which the cells were grown. As expected, the ICM are much more extensive when the cells are grown at LL. The quadruple Δ*puc*BA_abce_ mutant shows a broadly similar behaviour. The extent of the ICM under HL is rather less than the wild-type case but overall the shape and amount of the ICM is the same in the wild-type and Δ*puc*BA_abce_ cells .

**Fig. 4:**
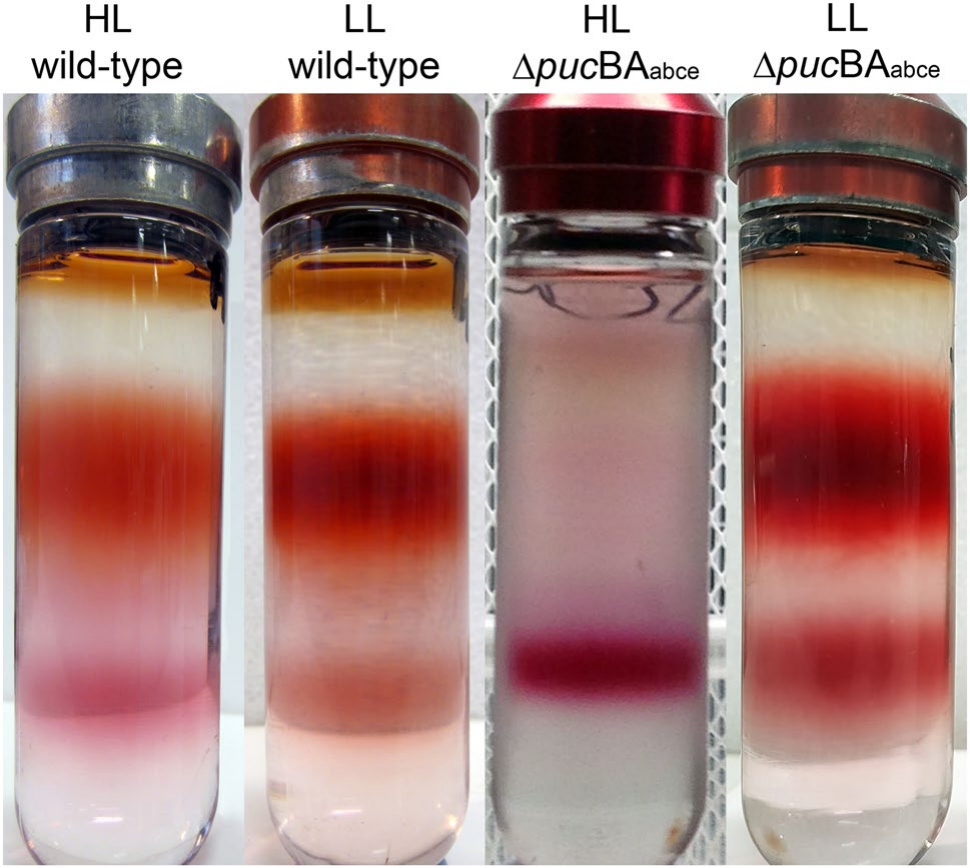
Fractionation of the photosynthetic units of solubilised membranes from HL and LL grown cells of both the wild-type and the quadruple mutant. The top pigmented band is the LH2 fraction and the lower pigmented band is the LH1-RC ‘core’ complex. It is evident that the PucD LH2 complex is not produced under HL conditions. The *brown*-coloured band right at the top of the gradients is due to free pigments

In order to demonstrate the phenotype of the Δ*puc*BA_abce_ mutant more clearly, membranes isolated from these cultures grown at HL and LL were solubilised and the LH complexes, i.e., LH2 and ‘core’ complexes were fractionated by sucrose density centrifugation. The results of these fractionations are presented in Fig. 4. The top strongly pigmented band evident in the gradients of the wild-type sample is the LH2 fraction and the bottom band is the ‘core’ complex fraction. The gradients from the wild-type sample show the normal response to light intensity with the ratio of LH2 to ‘core’ clearly being larger in the low-light case. Sucrose gradients prepared from the quadruple deletion mutant show a rather different behaviour. In this case, the gradient from the HL grown cells reveals that under this condition the mutant is unable to make LH2 and only the ‘core’ complex band is visible. The sucrose gradient from the LL grown deletion mutant shows that both LH2 and the ‘core’ complexes are made. In all cases the absorption spectra of the ‘core’ complex fractions are the same in the NIR region irrespective of the cell source and the growth conditions (data not shown). Interestingly, the carotenoids in the ‘core’ complex are different at HL and LL.

**Fig. 5:**
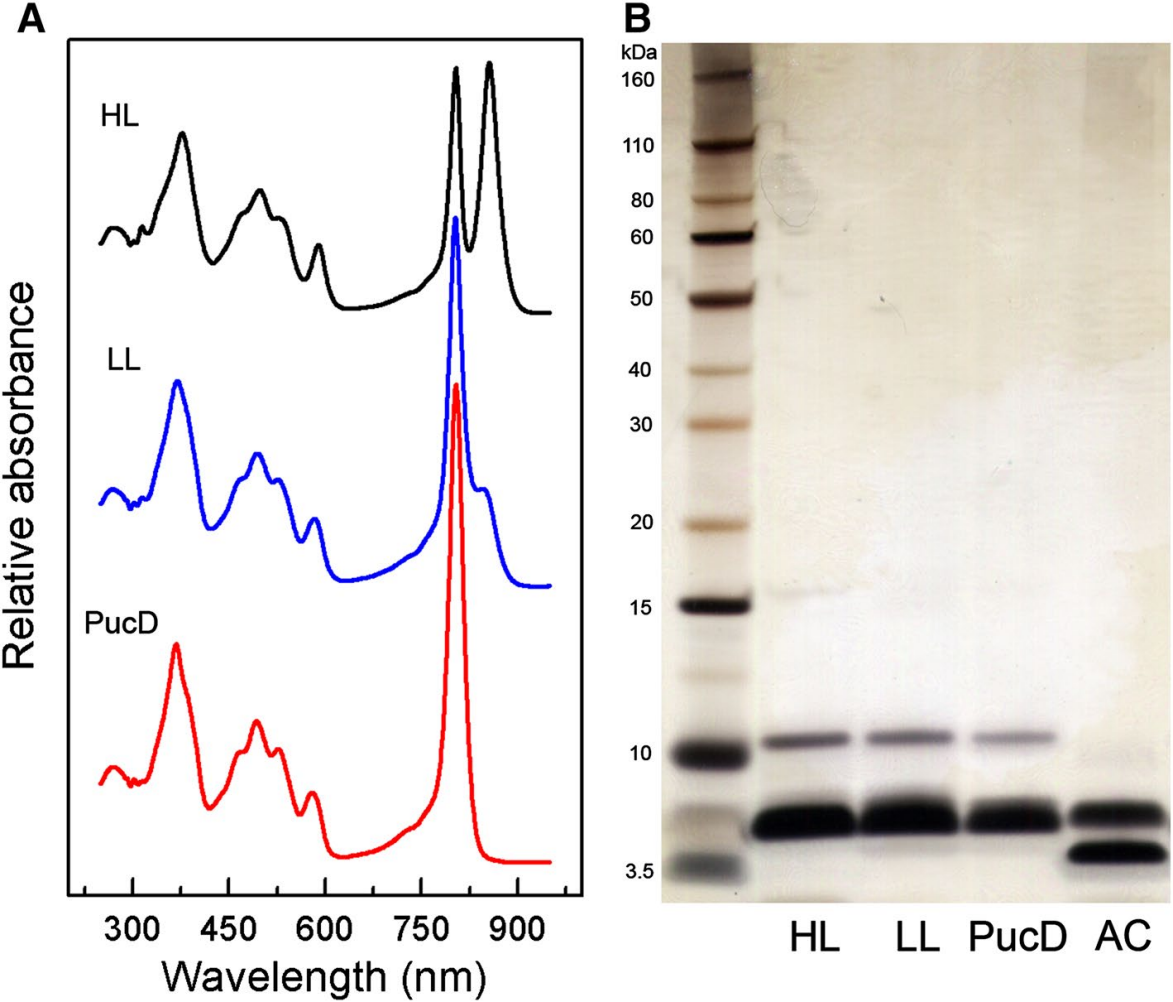
The absorption spectra of **a** the purified LH2 complexes and **b** a comparative analysis of their polypeptide composition by SDS-PAGE. AC is the purified LH2 from *Rbl. acidophilus* strain 10050. The ladder of bands down the left-hand side of the gel are standard molecular weight markers. The LH2 apoproteins are very hydrophobic and the apparent masses calculated from gel mobility do not reflect their true masses (see the data from the mass spectroscopic measurements detailed in the supplementary information)

The LH2 complexes from these gradients were further purified to homogeneity as described in the Methods with the subsequent room temperature absorption spectra presented in Fig. 5a. The wild-type LH2s show the typical change in the spectra on going from HL to LL conditions. In the NIR region both the HL and LL LH2 have a strong bacteriochlorophyll (Bchl) Q_y_ absorption band at 804 and 803 nm (B800) respectively, although the height of this band at LL is enhanced relative to the Bchl Q_x_ absorption peak at ~585 nm. The wild-type HL spectrum has a typical B850 band at 856 nm so that the NIR region of this sample is similar to the B800-850 LH2 complex from *Rbl. acidophilus* for which a high-resolution structure is available (McDermott et al. 1995; Papiz et al. 2003). The wild-type LL LH2 has a smaller, much less pronounced B850 band, the height and shape of which are very dependent on the precise light intensity at which the cells are grown. The LL LH2 complex from the quadruple Δ*puc*BA_abce_ mutant will be referred to as PucD LH2 from this point onwards as this complex only contains the transcribed and translated polypeptides from the *puc*BA_d_ gene pair. The LL PucD LH2 spectrum presented in Fig. 5a is immediately striking in that there is a complete absence of any B850 band. All the oscillator strength from the excitonically coupled ring of Bchls, which in the wild-type HL and LL samples absorbs at 856 and 849 nm, respectively, is now superimposed on the B800 band resulting in a much larger, single, narrow absorption band with a maximum at 804 nm. Figure 5b shows a SDS-PAGE gel of the purified LH2 samples in which their polypeptide composition is compared. Although the LH2 complexes in all cases are rather pure it is not possible just using this technique to distinguish their different polypeptide compositions. In order to confirm that the PucD LH2 does, in fact, only contain the α- and β-apoproteins encoded by the *puc*BA_d_ gene pair, the PucD LH2 was analysed by mass spectroscopy. The mass spectrum confirmed that only these two apoproteins were present (full data shown in Supplementary Fig. 1; Supplementary Table 4).

**Fig. 6:**
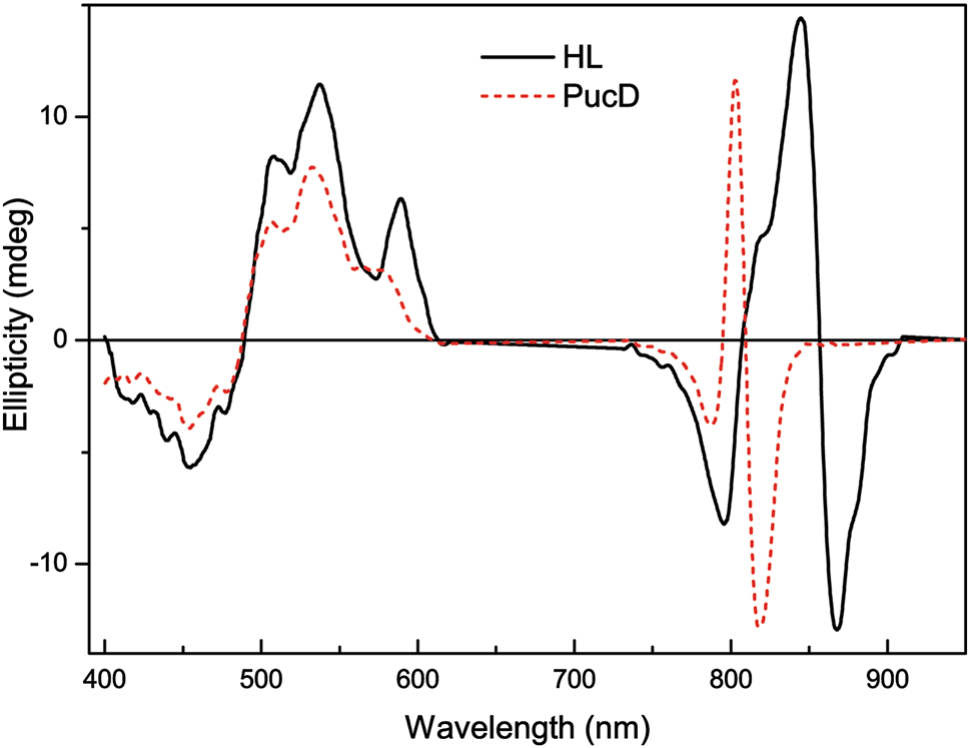
Visible and NIR CD spectra of the HL (*solid*) and PucD (*dash*) LH2 complexes. The LH2 complexes were dissolved in 20 mM Tris–Cl, 0.1% LDAO pH 8.0 and the CD spectra were run as described in the “Materials and methods” section above

Since the PucD LH2 complex only contains a single absorption band at 804 nm it is important to investigate whether the basic arrangement of the Bchls in this complex is similar to, or different from, that seen in the wild-type HL LH2 complex. A CD spectrum of purple photosynthetic antenna complexes is able to provide detailed information on the organisation and interactions of the optically active pigments, in this case the Bchl pigment molecules within the LH2 ring. The visible and NIR CD spectra for wild-type HL and PucD LH2 complexes are presented in Fig. 6. The wild-type HL CD spectrum in the NIR shows a doublet band in the region of the B850 absorption band that overlaps with a second weaker doublet in the region of the B800 absorption band. The HL spectrum is consistent with that previously recorded by van Mourik et al. (1992) with a strong doublet band and a negative band at 797 nm. The CD spectrum of the PucD LH2 complex also shows a strong doublet band of about the same relative intensity as that seen in the case of the wild-type HL LH2 complex but shifted to the blue and a weak negative band at about 787 nm. These changes in the CD spectrum in the case of the PucD LH2 appear to simply reflect a blue shift of the ‘B850’ doublet band seen in the HL LH2 complex. This suggests that the basic exitonic structure of the LH2 complex from the PucD only mutant is very similar to that seen in the HL LH2 complex.

When comparing the properties of the PucD LH2 with those of the wild-type HL LH2 complex, it is also interesting to check whether or not they have the same ratio of carotenoids to Bchls. All types of LH2 so far investigated have a bacteriochlorophyll to carotenoid ratio of 3:1, for example, see Cogdell and Crofts (1978). The total pigments present in 2–4 replicates of four different preparations of the purified PucD LH2 complex were extracted into 7:2 (v/v) acetone:methanol as described in the Methods. The ratio of the bacteriochlorophyll absorption (*A*_max_ = 772 nm) with that of the carotenoid bands (*A*_max_ = 476 − 78 nm) was determined and compared with an equivalent number of samples for wild-type *Rps, palustris* HL and LL LH2 complexes, see Table 1. It is apparent that these ratios are the same, within experimental error, and suggests that the basic structure of the novel PucD LH2 complex is similar to both the HL and LL LH2 complexes from *Rps. palustris* and, indeed, has a similar pigment composition to the well-studied, wild-type B800-850 LH2 complex from *Rbl. acidophilus* (McDermott et al. 1995).

**Table 1:** The Bchl:carotenoid ratio in the three types of LH2 complexes

LH2	Ratio (A_772_/A_478_)	Molar ratio^a^
HL	1.44 ± 0.09	2.99 ± 0.18
LL	1.51 ± 0.06	3.17 ± 0.13
PucD	1.47 ± 0.10	3.09 ± 0.21

^a^The true value of the molar extinction coefficient for the *Rps. palustris* LH2 carotenoids in this solvent mixture at 478 nm is unknown. Nevertheless, the value chosen is a reasonable approximation

Ultimately the only way to really understand how the structure of the PucD LH2 complex produces its unusual NIR absorption band is to determine that structure by X-ray crystallography. To this end crystallisation trials with this LH2 complex have been undertaken. Initially several trials with sparse-matrix crystallisation screens were set up using a HT-96 well format as described in the Methods. These trials produced several crystal ‘hits’ and after promising initial diffraction testing using our home X-ray source, Condition 41 (44% PEG 600, 50 mM CaCl_2_, 20 mM Tris–Cl) from screen MemGold was selected for further optimisation.

**Fig. 7:**
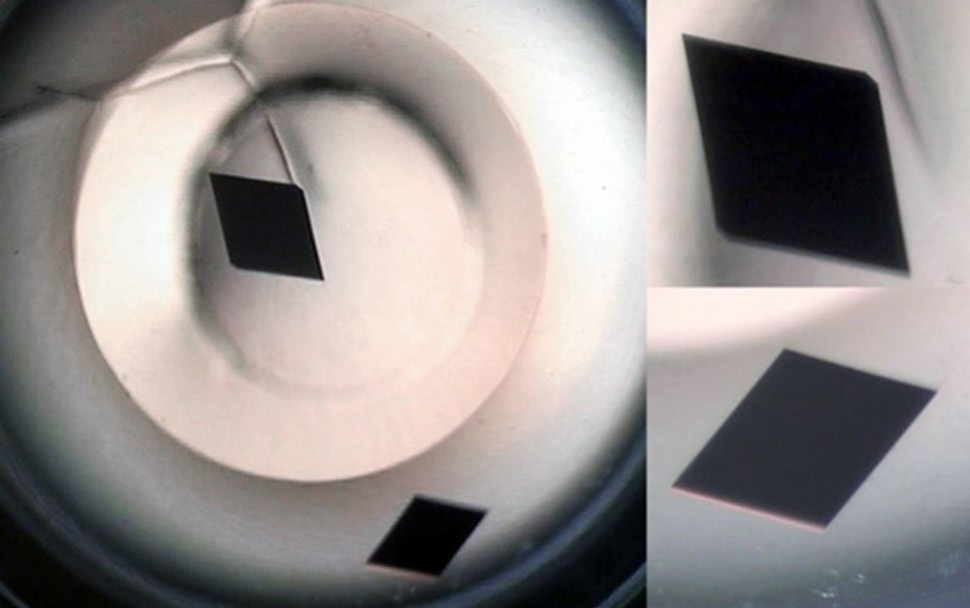
Typical crystals grown following optimisation of the initial conditions identified by sparse-matrix screening. These crystals have a length in the longest dimension of ~700 µm

In general, the intensity of diffraction becomes stronger as the number of molecules in the crystal increases and weaker as the size of the crystallised complex increases. In addition, crystals of membrane proteins, such as the LH2 complex, contain a very high percentage of solvent (~70%) that includes the disordered detergent. This means that large well-ordered crystals are required to obtain the strongest as possible X-ray diffraction intensities. To grow bigger crystals, it was necessary to upscale the trials to larger 24-well plates. After several rounds of optimisation, the crystallisation conditions given in the Methods were found to consistently produce large, sharp-edged, well-formed crystals, as shown in Fig. 7. Several sets of X-ray diffraction data were collected for these crystals at the DLS synchrotron where diffraction spots were observed up to 4.4 Å resolution. The data were processed by programs *XDS* (Kabsch 2010), *POINTLESS* (Evans 2006) and *AIMLESS* (Evans and Murshudov 2013) and subsequently reduced by the automated data reduction pipeline *xia2* (Winter 2010) and other programs from the CCP4 suite (Winn et al. 2011). Table 2 lists processing statistics for the best diffracting crystal at the final resolution cut off at 4.71 Å.

**Table 2:** Diffraction data processing, molecular replacement and refinement statistics for the PucD LH2 crystal

Data processing	
Space group	P2_1_
Cell dimensions	
*a, b, c* (Å)	97.73, 127.18, 169.27
*α, β, γ* (º)	90.0, 90.42, 90.0
Solvent contents (%)	~70
Beamline	I03 at DLS
Wavelength (Å)	0.9710
Resolution range (Å)	97.73–4.71
Highest-resolution shell (Å)	4.83–4.71
No. of unique reflections^a^	21,084 (1545)
Redundancy	3.4 (3.3)
Completeness (%)	97.5 (98.0)
Mean *I*/*σ* (I)	10.5 (2.3)
*R*_merge_^b^ (%)	4.6 (59.7)
Molecular replacement figures of merit	
Rotation function Z-score (RFZ)	5.5
Translation function Z-score (TFZ)	25.4
Log-likelihood gain (LLG)	884
Jelly-body refinement	
Resolution range (Å)	97.73–4.71
*R*_work_/*R*_free_^c^ (%)	40.09/42.69
Number of reflections used	19,903
Number of R_free_^c^ reflections	1167

^a^Values in parentheses refer to the highest-resolution shell

^b^ *R*_merge_ = Σ_*hkl*_ Σ_*i*_|*I*_*i*_(*hkl*) − <*I*(*hkl*)>|/Σ_*hkl*_ Σ_*i*_ *I*_*i*_(*hkl*)

^c^ *R*_work_ and *R*_free_ = Σ_*hkl*_ ||*F*_o_(*hkl*)| −|*F*_c_(*hkl*) ||/Σ_*hkl*_|*F*_o_(*hkl*); *R*_work_ was calculated for all data except for 5.5% that was used for the *R*_free_ calculations

The molecular replacement (MR) program *Phaser* (McCoy et al. 2007) was then applied to find the solution of the PucD LH2 crystal structure using the 4.71 Å data with the nonameric *Rps acidophila* 10050 LH2 structure (McDermott et al. 1995) as a search model (see the figures of merit for this solution in Table 2). When the full model of *Rps acidophila* 10050 LH2 (McDermott et al. 1995) was used *Phaser* produced nine solutions with the Log-Likelihood Gain (LLG) values between 852 and 858, exceeding significantly the expected LLG (eLLG) target value of 225. Translation function Z-score (TFZ) for these solutions was in a range of 26.1–26.5, much greater than a benchmark value of 8.0 suggesting a correct solution, therefore, indicating that the definite solution with the correct packing has been found. Each solution comprised two *Rbl. acidophilus* LH2 complexes forming a tail-to-tail dimer (‘tail’ being the C-terminus of the LH2 polypeptides). In these nine solutions one ring is identical while the second ring is related by the nine-fold symmetry, i.e. by a multiple rotation of 40°, about the *Rbl. acidophilus* LH2 ring axis, so effectively these nine solutions are identical. Similar dimers were formed about the twofold axis in the crystal structure of *Rps acidophila* 10050 LH2 in spite of completely different crystal symmetry (space group *H*32).

**Fig. 8:**
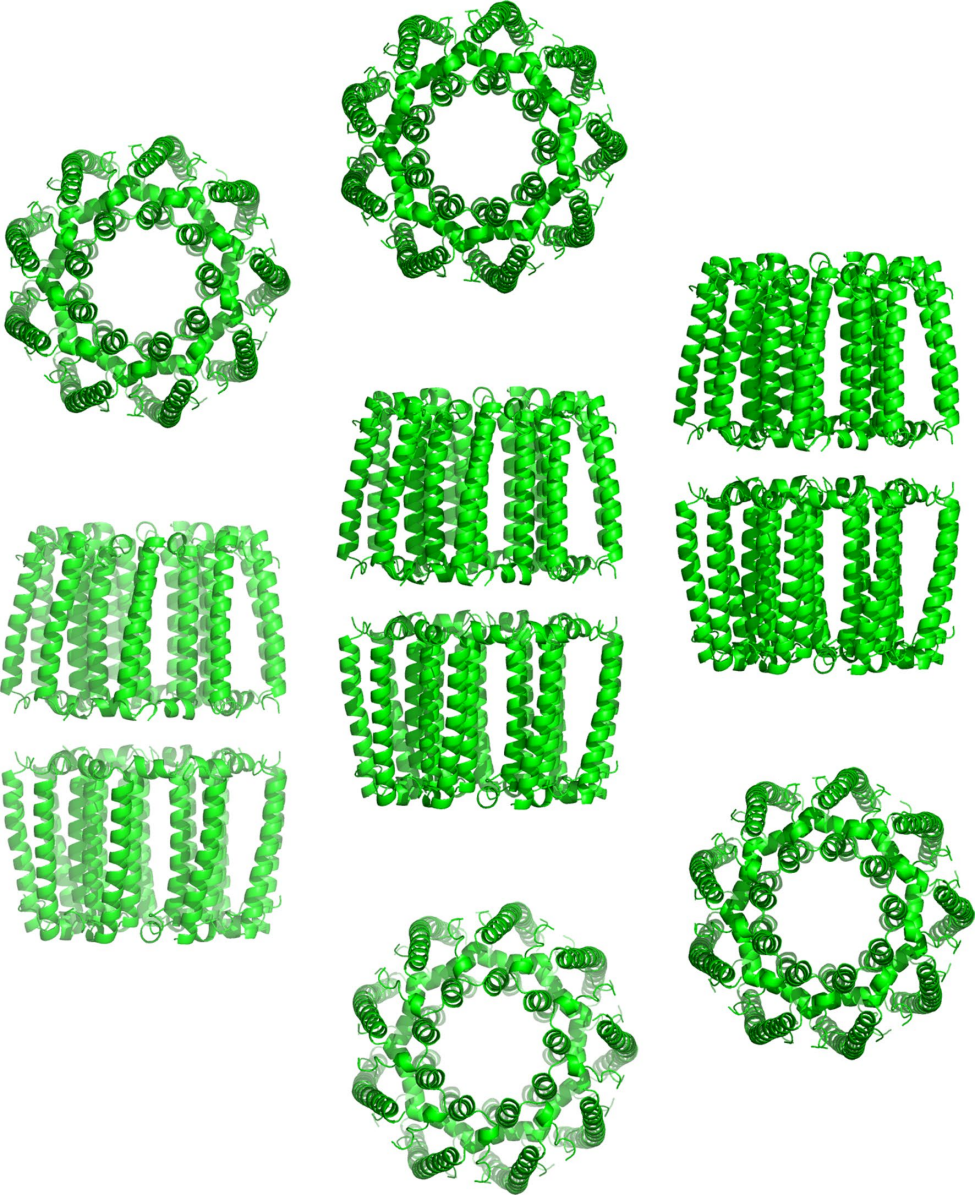
The packing arrangement of the PucD LH2 molecules in the crystal determined by molecular replacement. The pigments have been omitted for clarity

As this MR solution included some clashes at the C-termini (almost 3% of the trace atoms), the *Rps acidophila* 10050 LH2 model has been pruned to remove the C-termini sticking out of the LH2 ring. This pruned acLH2 model was subsequently used as an MR model and produced a single *Phaser* solution without any clashes and with a slightly improved LLG value of 884 and TFZ value of 25.4. The dimeric tail-to-tail arrangement and the crystal packing of this solution was the same as that discussed above. The packing of dimers of PucD LH2 in the crystal lattice is shown in Fig. 8.

Attempts were also made to use octameric models of the LH2 complex as MR models. A theoretical octameric model based on the *Rbl. acidophilus* LH2 structure produced 12 dimeric solutions with some allowable clashes at the C-termini, however, these solutions were all with LLG values (maximum of 152) lower than the eLLG target value of 225. Moreover, these 12 solutions exhibited a variety of rotations about the LH2 ring axis suggesting that a single octameric arrangement is not in agreement with the experimental diffraction data. Electron density maps for these octameric solutions were also of much lower quality than the maps for the nonameric *Rps acidophila* 10050 LH2-like solutions. Finally, 1934 ‘solutions’ were produced by *Phaser* when the octameric *Phaeospirillum (Psp.) molischianum* (formerly *Rhodospirillum (Rsp.) molischianum*) LH2 (Koepke et al. 1996) model was used as an MR model. Although the top solutions had LLG values up to 144 (again, below the eLLG target value of 225) their two components, the two LH2 rings, were in a physically impossible side-to-tail arrangement with many disallowed clashes. The *Phaser* runs for the octameric MR models required extremely long CPU times suggesting a very poor agreement with the experimental data. All the *Phaser* figures of merit and the CPU times for all four MR models are shown in Supplementary Table 5. Altogether, these MR tests confirmed that the PucD LH2 complex has nonameric symmetry.

Finally, the best nonameric MR solution was refined using ‘jelly-body’ restraints with the program *REFMAC5* (Murshudov et al. 2011). After 90 cycles of this refinement the *R*/*R*_free_ factors were 40.09/42.69, again indicating correctness of the overall features of the obtained crystal structure for the PucD LH2. Both *Phaser* and *REFMAC5* electron density maps are quite satisfactory for the α-polypeptides but suggest that the β-polypeptides for the PucD LH2 complex are likely to be tilted slightly relative to the orientation observed in the *Rbl. acidophilus* LH2 complex. Similarly, electron density for the 18 macrocycles of the B850 Bchls is relatively decent for this low resolution, whereas the quality of electron density for the nine B800 Bchls suggests that these pigments in the PucD LH2 complex are likely to be in a different orientation than in the *Rbl. acidophilus* LH2 complex. Unfortunately, these maps are of limited quality and don’t allow the refinement of the details of the PucD LH2 structure. This is due to both the limited resolution of diffraction data and the likely inadequacy of the MR model (the *Rps acidophila* 10050 LH2 complex) used for the molecular replacement for the PucD LH2 data, hence the relatively low quality phasing.

High-resolution diffraction data should allow better electron density maps to be obtained and eventual remodelling of the structure, even with this less than adequate MR model. We are, therefore, continuing to try to obtain crystals with improved diffraction.

## Discussion

The Δ*puc*BA_abce_ mutant shows two interesting and usual phenotypes. Firstly, the Δ*puc*BA_abce_ mutant only makes PucD LH2 at LL intensities. In other words, the Δ*puc*BA_abce_ mutant grown at our HL conditions makes only ‘core’ complexes. There are at least two possibilities for this; either at HL the *puc*BA_d_ gene pair is expressed but the PucD LH2 is not assembled or the *puc*BA_d_ gene pair is simply not expressed. This latter possibility seems the most likely since mass spectrometry analyses performed by Brotosudarmo et al. (2011) on wild-type cells found that at HL the LH2 complex was composed of polypeptides only from the *puc*BA_a_ and *puc*BA_b_ gene pairs, whereas at LL the LH2 was composed of primarily polypeptides from the *puc*BA_d_ gene pair with relatively little from the *puc*BA_a_ and *puc*BA_b_, gene pairs. Future work will test for the activity of the promoter from the *puc*BA_d_ operon to see if production of mRNA from this operon only occurs when the cells of the Δ*puc*BA_abce_ mutant are grown at LL.

The second interesting and usual phenotype is that the purified PucD LH2 complex has a rather unique NIR absorption spectrum. This then raises the question as to whether this complex has a similar or different structure when compared with the standard wild-type HL LH2 complex. The *puc*BA gene sequences are shown in Supplementary Fig. 1. The sequences are all rather homologous and are similar to *puc*BA genes from other species of purple photosynthetic bacteria. The lack of high-resolution crystal structures from LH2 complexes with different absorption spectra means that it is not clear how to conclude the presence of which amino acids produce which spectroscopic form. Previous studies have highlighted the role of hydrogen bonds from two alpha-polypeptide aromatic residues that correlate with the strongly coupled Bchls absorbing at 850 nm (McDermott et al. 1995). If these residues change to non-hydrogen bonding residues, such as FM, then the 850 nm absorption band is usually blue shifted (Brunisholz and Zuber 1992; Fowler et al. 1992; McLuskey et al. 2001). In the case of the *puc*BA_d_ gene pair this pair of residues are F and M. However, the details of the molecular interactions that induce the ‘B850’ band to blue shift all the way to 803 nm are not yet clear.

The CD spectra and the Bchl:carotenoid ratios of these two types of LH2 complex suggest that they both have rather similar overall structures. Of course, to finally prove this will require a good, high-resolution structure of the PucD LH2 complex. Unfortunately, so far it has only been possible to grow crystals of the PucD LH2 complex that are not well ordered enough to allow such a high-resolution X-ray crystal structure to be determined. These efforts to obtain better crystals are continuing along with trials using completely different crystallisation methodology based on cubic lipidic phases (Caffrey 2009).

One interesting feature of the NIR absorption spectrum of the PucD LH2 complex is the intensity of the 804 nm band. If this band arises from just a blue shift of the B850 absorption band seen in the wild-type HL LH2 complex, it might have been expected that the blue-shifted band would be broader than the PucD LH2 B800 band seen in Fig. 5a (in fact, at half height the width of the B850 band is ~ 20% greater than the B800 band in the PucD LH2 complex). The increase in intensity of the 800 nm band is not just due to the superpostition of the two Bchl Q_y_ absorption bands, rather it is also enhanced by the narrowing of that portion of the absorption band that reflects the absorption due to the ring of tightly coupled Bchl molecules. This suggests that the PucD LH2 complex in this absorption band exhibits reduced inhomogeneous broadening relative to that seen in the case of the wild-type LH2 complex.

The PucD LH2 absorption spectrum presented in Fig. 5a with a single strong NIR absorption band at 804 nm is similar, although narrower, to the absorption spectrum of the ‘LH4’ complex described previously by Papiz and co-workers (Hartigan et al. 2002). This complex was obtained from wild-type *Rps. palustris* grown at very low-light intensities. However, our group has never been able to reproduce this spectroscopic form under our growth conditions and the Δ*puc*BA_abce_ deletion mutant membrane spectra are more reminiscent of the B800 LH2 found in *Rhodobacter denitrificans* (Duquesne et al. 2011).

This paper illustrates the power of a ‘genetic dissection’ approach to purple bacterial species that have a multigene family of *puc* genes. Through this approach it is possible to unequivocally determine the *puc* genes that are responsible for any spectroscopic form of LH2.

## Electronic supplementary material

Below is the link to the electronic supplementary material.

Supplementary material 1 (DOCX 2919 KB)

## Abbreviations

Bchl: Bacteriochlorophyll
CD: Circular dichroism
DLS: Diamond light source
DM: *n*-Decyl-β-d-maltoside
DDM: *n*-Dodecyl-β-d-maltoside
HPT: Heptanetriol
HL: High light
ICM: Intracytoplasmic membranes
LDAO: *N,N*-Dimethyldodecylamine *N*-oxide
LH: Light-harvesting
LLG: Log-likelihood gain
RC: Reaction centre
LL: Low light
MR: Molecular replacement
NIR: Near infra-red
OD: Optical density
Psp.: Phaeospirillum
PSU: Photosynthetic units
*Rba*: *Rhodobacter*
*Rbl*: *Rhodoblastus*
*Rps*: *Rhodopseudomonas*
Rsp: Rhodospirillum
RFZ: Rotation function Z-score
TFZ: Translation function Z-score
TEM: Transmission electron microscopy
WT: Wild-type
