# Supplementary material for: Characterisation of a pucBA deletion mutant from Rhodopseudomonas palustris lacking all but the pucBAd genes
Source: Photosynth Res. 2017 May 31;135(1):9–21. doi: 10.1007/s11120-017-0386-7 (PMC5783997; doi:10.1007/s11120-017-0386-7)
Supplement: Supplementary file 1 — Supplementary material 1 (DOCX 2919 KB) [file 11120_2017_386_MOESM1_ESM.docx]

Supplementary Figure 1.


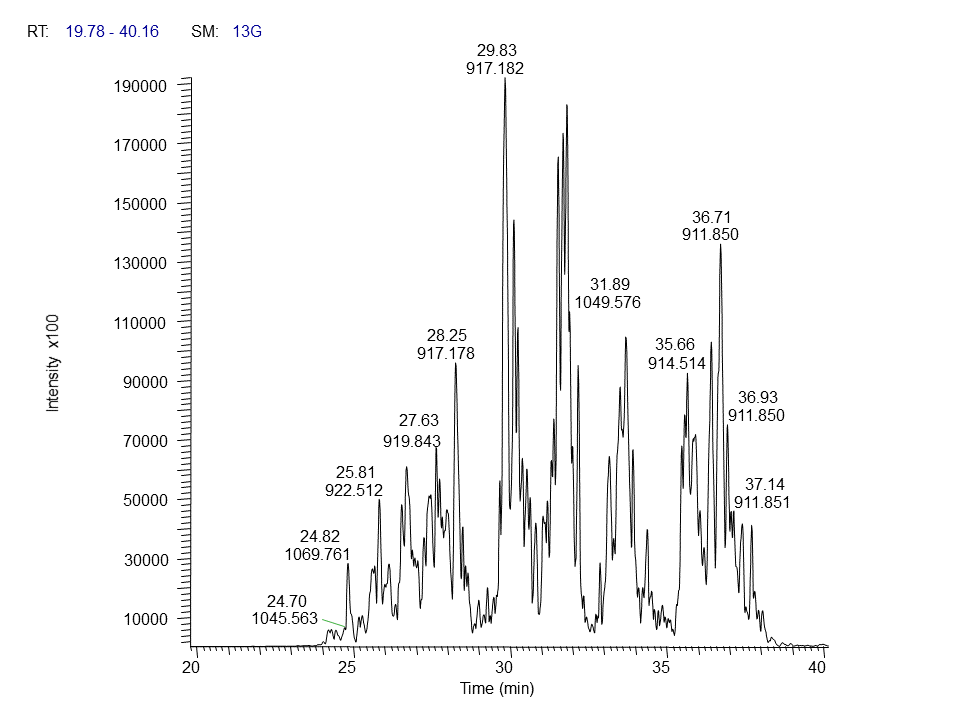


A plot of the signal intensity (base peak chromatogram) from the liquid chromatography-mass spectrometric (LC-MS) analysis of PucD LH2 complex. The X axis is the retention time of the peptides eluting from the chromatographic system and the Y axis is the signal intensity. The figures above the peaks are the retention time of each peak (upper) and the mass to charge ratio (m/z) of the peptide.

Supplementary Table 1.

| *puc*A_a_ | 2690946 | *puc*B_a_ | 2690607 |
| --- | --- | --- | --- |
| *puc*A_b_ | 2689917 | *puc*B_b_ | 2690915 |
| *puc*A_c_ | 2691622 | *puc*B_c_ | 2688839 |
| *puc*A_d_ | 2690955 | *puc*B_d_ | 2690920 |
| *puc*A_e_ | 2692095 | *puc*B_e_ | 2690556 |

The Genbank numbers are listed for the *puc*BA gene pairs from *Rps palustris* CGA 009 chromosome NCBI Reference Sequence NC_005296.

Supplementary Table 2.

| Gene | Forward | Reverse |
| --- | --- | --- |
| *puc*BA_a_ upstream | atgagaattcctaaggtcgagctggaggtg | atcaggatccgaagtactggaacggcaagg |
| *puc*BA_a_ downstream | attaggatcccgagtaaacgtaggcgagga | catagcatgcatgcgccttactcgtcagatc |
| *puc*BA_c_ upstream | atgagaattccaatcgacgctacgcctatac | atcaggatccttgtggatttcctcggattc |
| *puc*BA_c_ downstream | attaggatccccgctttcatgaacggtaag | catagcatgcgcagtgttttcagccatcac |
| *puc*BA_b_ | atcggcatgcgataatgtgactgcgcgaga | tgcaggatccttccaatttcgagcaaatcc |
| Streptomycin resistance  gene | tcaaccggtcgagattttcaggagctaagga | ctaaagtacttttctaggcaccaataactgc |
| *puc*BA_e_ | aaaggcatgcgccgaccgcgacctgaaaca | aatacgggccccgtacgccgagggctggaa |
| Chloramphenicol reistance gene | gataaccggtagattttcaggagctaagga | ctaacctggtttctaggcaccaataac |

The primer sequences used to make the respective *puc*BA gene pair deletions.

Supplementary Table 3.

| Gene | Forward | Reverse | Wild Type  *puc*BA bp | Recombinant  *puc*BA bp |
| --- | --- | --- | --- | --- |
| *puc*BA_a_ check | ttcggctgacctggtatctc | ggttgcagaggaagattgg | 1498 | 1168 |
| *puc*BA_b_ check | caccgtctgtccgttctgta | gctgtcatgaatcgcaaaaa | 1047 | 1661 |
| *puc*BA_c_ check | cagaacgaattcggcttagg | ggactcggaaatccttcctc | 1280 | 930 |
| *puc*BA_d_ check | gtcgcgtgagtttctgatcc | tcgacgatcggattgtacttc | 1246 | - |
| *puc*BA_e_ check | tcactcggatgtgatcagga | aaagatcaaaggcagcgaaa | 1014 | 1488 |

Primer sequences used to check the *puc*BA gene deletions and the expected sizes of the PCR amplified gene products in the case of the wild-type strain and the quadruple deletion mutant (see Figure 1).

Supplementary Table 4.

| M/Z | M+1 | RT | Sequence | Oxidised residue | Peptide |
| --- | --- | --- | --- | --- | --- |
| 829.581 | 5801.023 | 28.18 | MVDDPNKVWPTGLTIAESEELHKHVIDGSRIFVAIAIVAHFLAYVYSpWLH | P48 | *puc*B_d_ |
| 916.677 | 5495.026 | 28.10 | MNQGRIWTVVKPTVGLPLLLGSVAImVFLVHFAVLTHTTWVAKFmNGKAA | M26, M45 | *puc*A_d_ |
| 788.153 | 5511.024 | 27.71 | mNQGRIWTVVKPTVGLPLLLGSVAImVFLVHFAVLTHTTWVAKFmNGKAA | M1, M26, M45 | *puc*A_d_ |
| 943.167 | 5653.965 | 38.59 | VDDPNKVWPTGLTIAESEELHKHVIDGSRIFVAIAIVAHFLAYVYSPWLH |  | *puc*B_d_ |
| 965.010 | 5785.021 | 31.95 | MVDDPNKVWPTGLTIAESEELHKHVIDGSRIFVAIAIVAHFLAYVYSPWLH |  | *puc*B_d_ |
| 1045.778 | 5224.861 | 38.98 | mNQGRIWTVVKPTVGLPLLLGSVAIMVFLVHFAVLTHTTWVAKFmNG | M1, M45 | *puc*A_d_ |
| 1069.096 | 6409.539 | 24.67 | mNQGRIWTVVKPTVGLPLLLGSVAImVFLVHFAVLTHTTWVAKFmNGKAAAIESSIKAV | M1, M26, M45 | *puc*A_d_ |

Table Y. Displays the m/z ratio of the peptides eluted at each retention time (RT), along with their mass (M+1) and the sequence of amino acids, including oxidised residues, obtained by analysis of the MS fragmentation data in Proteome Discoverer.

Supplementary Table 5.

| MR model | AC LH2 (full size)  nonameric | AC LH2 (pruned)  nonameric | AC LH2 (theoretical  octameric | *Rps molischianum*  LH2, octameric |
| --- | --- | --- | --- | --- |
| RFZ^a^ | 6.4 | 5.5 | 3.7 | 3.8 |
| TFZ^b^ | 26.5 | 25.4 | 10.6 | 6.0 |
| PAC^c^ (%) | 3, clashes at the  C-termini contacts | 0 | 1-10, clashes at  the C-termini | 8, types of clashes  disallowed |
| LLG^d^ | 858 | 884 | 152 | 144 |
| eLLG^e^ | 225 | 225 | 225 | 225 |
| No. of solutions  with description | 9 dimeric, almost  identical solutions  related by 40^o^ rotation | 1 dimeric solution  without clashes,  LH2 ring model is shorter along the  main ring axis and  the C-termini are removed | 12 dimeric solutions  with various  non-40^o^ ring axial  rotations to mimic  non-octameric  symmetry | 1934 solutions  with disallowed  clashes, two rings  in pseudo-dimers  form side-tail  clashes |
| CPU time  (minutes) | 11 | 9.5 | 646 | 1867 |

^a^ RFZ: Rotation Function Z-score; the score to judge the signal-to-noise of the peak in the map;

^b^ TFZ: Translation Function Z-score, meaning as above, the higher value than 8 for definite solution;

^c^ PAC: crystal packing in %, solutions accepted if pairwise clashes less than 10% of trace atoms;

^d^ LLG: Log-Likelihood Gain, the maximum likelihood is the probability that the diffraction data would have been measured, given the MR model, so it allows to compare how well different models agree with the data;

^e^ eLLG: expected LLG judged by *Phaser.*

Various statistics and descriptive details for four different molecular replacement (MR) models of the *Rps palustris* PucD LH2 obtained from the latest version of the *Phaser* program (2.7.17).

**Supplementary Figure 1.**

**
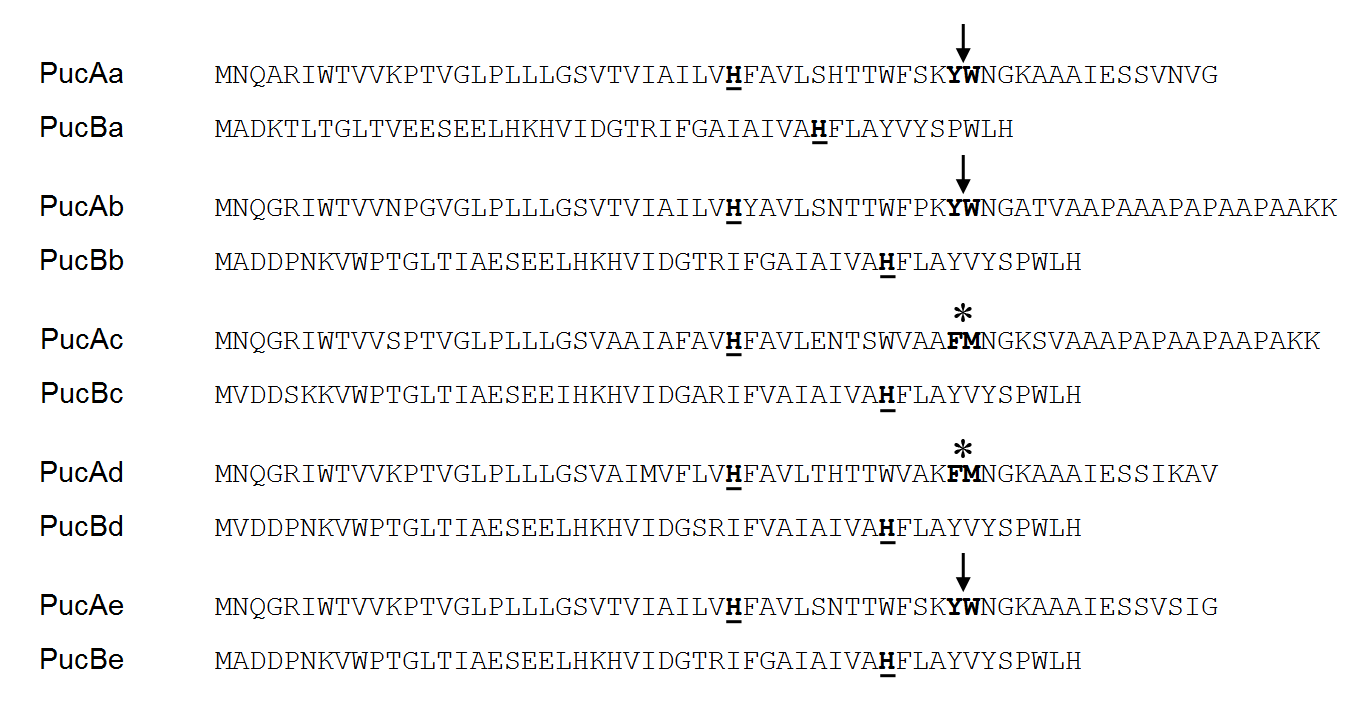
**

Primary sequences are shown of the α- and β-polypeptides respectively from the five *puc*BA gene pairs described in the manuscript (Larimer et al 2004). The *puc*BA_c_ gene pair is presumed to be pseudogenes. In each polypeptide, a conserved Histidine residue is underlined that is presumed to form a ligand to the central Mg^2+^ ion of the strongly coupled B850 ring in the HL LH2 complex from *Rps. acidophila* (McDermott et al 1995). Each α-polypeptide is also marked with either an arrow or an asterisk. The arrow illustrates the aromatic amino acid doublet that correlates with the rhift of the ring to around 850 nm. The α-polypeptides marked with an asterisk do not have this aromatic amino acid doublet and, therefore, are presumed not to be able to induce such a pronounced red-shift.
